# Postoperative Prognostic Nutritional Index as a Useful Prognostic Factor in Patients With Gastric Cancer

**DOI:** 10.1002/ags3.70057

**Published:** 2025-06-19

**Authors:** Masaaki Yamamoto, Takeshi Omori, Yasunori Masuike, Naoki Shinno, Hisashi Hara, Takahito Sugase, Takashi Kanemura, Atsushi Takeno, Motohiro Hirao, Hiroshi Miyata

**Affiliations:** ^1^ Department of Gastroenterological Surgery Osaka International Cancer Institute Osaka Japan; ^2^ Department of Surgery NHO Osaka National Hospital Osaka Japan; ^3^ Department of Gastroenterological Surgery, Osaka International Medical and Science Center Osaka Keisatsu Hospital Osaka Japan; ^4^ Department of Surgery Toyonaka Municipal Hospital Toyonaka, Osaka Japan; ^5^ Department of Gastroenterological Surgery Sakai City Medical Center Sakai City, Osaka Japan

**Keywords:** biomarker, gastric cancer, postoperative, prognostic nutritional index, surgery

## Abstract

**Aim:**

To verify whether postoperative prognostic nutritional index is a useful prognostic factor in patients with gastric cancer.

**Methods:**

This study included 1738 consecutive patients with gastric cancer who underwent radical gastrectomy at our institution from January 2004 to December 2018. The sensitivity and specificity of white blood cell, neutrophil, lymphocyte, monocyte, and platelet counts, C‐reactive protein, hemoglobin, and albumin levels, neutrophil‐to‐lymphocyte ratio, lymphocyte‐to‐monocyte ratio, C‐reactive protein‐to‐albumin ratio, platelet‐to‐lymphocyte ratio, and prognostic nutritional index on postoperative Days 1 and 3 in predicting recurrence were evaluated using receiver operating characteristic curves. Recurrence‐free survival and overall survival were compared between the normal and high fibrinogen groups.

**Results:**

After applying the inclusion criteria, 1635 eligible patients were included in the analysis. The prognostic nutritional index on postoperative Day 1 attained the highest area under the curve (0.699). Overall survival and recurrence‐free survival in the low prognostic nutritional index on postoperative Day 1 group were significantly poorer than those in the high prognostic nutritional index on postoperative Day 1 group (log‐rank test, both *p* < 0.001). Multivariate Cox analysis revealed that prognostic nutritional index on postoperative Day 1 was a significantly independent prognostic factor for overall survival and recurrence‐free survival (*p* = 0.002 and *p* < 0.001, respectively).

**Conclusion:**

Postoperative prognostic nutritional index was a useful prognostic factor in patients with gastric cancer.

## Graphical abstract

In patients after gastric cancer surgery, low postoperative PNI values, considering nutritional status, surgical stress, and postoperative inflammatory response, were significantly associated with poor prognosis in terms of both RFS and OS.

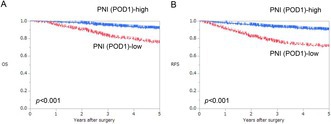

## 1 Introduction

Gastric cancer ranks as the fifth most common malignancy and the fifth leading cause of cancer death globally [1]. Various prognostic markers based on blood tests have been demonstrated by several studies to predict postoperative recurrence in patients with gastric cancer. Among them, the preoperative prognostic nutritional index (PNI) reflects the preoperative nutritional status and is reportedly useful in predicting postoperative complications and prognosis in various types of cancers, including gastric cancer [2, 3, 4]. Increased postoperative inflammatory response, represented by postoperative C‐reactive protein (CRP) levels, is associated with postoperative complications and prognosis [5, 6]. Nonetheless, preoperative PNI does not reflect the impact of surgical stress or postoperative complications, and postoperative CRP levels do not take the preoperative nutritional status into account, making them insufficient as prognostic markers. To the best of our knowledge, no previous study has evaluated prognostic markers that simultaneously consider preoperative nutritional status, surgical stress, and postoperative inflammation in patients with cancer. As the PNI is calculated from albumin levels and lymphocyte count, it can be considered not only an indicator of nutritional status but also a marker reflective of surgical stress.

Therefore, the current study aimed to investigate whether postoperative PNI is useful in predicting prognosis. We hypothesized that the postoperative PNI, which reflects not only the nutritional status at the time of surgery but also the impact of surgical stress and postoperative complications, could be a more useful prognostic factor.

## 2 Methods

### 2.1 Patients

This retrospective study included consecutive patients who underwent radical gastrectomy for gastric cancer at the Osaka International Cancer Institute from January 2004 to December 2018. The exclusion criteria were as follows: patients who had pStageIV or underwent R1/R2 resection or chemotherapy prior to gastrectomy.

Tumor status (TNM staging) was determined based on the 15th edition of the Japanese Classification of Gastric Carcinoma [7], which is equivalent to the 8th edition of the Union for International Cancer Control's TNM classification [8]. Postoperative complications were assessed using the Clavien–Dindo classification [9]. PNI was calculated using the following equation: [(10× serum albumin (g/dL) + (0.005× total lymphocyte count))] [2]. White blood cell (WBC), neutrophil, lymphocyte, monocyte, and platelet counts as well as CRP, hemoglobin, and albumin levels were examined within 30 days prior to surgery and on postoperative day (POD) 1 and POD3. Neutrophil‐to‐lymphocyte ratio (NLR), lymphocyte‐to‐monocyte ratio (LMR), CRP‐to‐albumin ratio (CAR), and platelet‐to‐lymphocyte ratio (PLR) were determined from blood collection data on POD 1 and POD 3. PNI was calculated using blood collection data within 30 days prior to surgery and on POD 1 and POD 3.

The protocol for this research project conformed to the provisions outlined in the Declaration of Helsinki and was approved by the Ethics Review Committee of Osaka International Cancer Institute (approval number #23153). Informed consent was obtained from all participants.

### 2.2 Postoperative Management

Patients were routinely followed up at 3–6 months postoperatively based on the 5th edition of the Japanese Gastric Cancer Treatment Guidelines [10].

### 2.3 Statistical Analysis

The sensitivity and specificity of PNI in predicting recurrence were compared to those of other biomarkers on POD1 and POD3 using receiver operating characteristic (ROC) curves. Associations between clinicopathological factors and the PNI on the POD1 group were analyzed using the chi‐square test for categorical variables and the Mann–Whitney *U* test for continuous variables. Recurrence‐free survival (RFS) was defined as the period from the date of surgery to the date of the first recurrence or death from any cause. Overall survival (OS) was defined as the period from the date of surgery to the date of death from any cause. The Kaplan–Meier method was employed for survival analysis, and differences in survival were tested using the log‐rank test. All statistical comparisons were two‐sided, with statistical significance set at *p* < 0.05. All statistical analyses were performed using JMP Pro software version 17.0.0 (SAS Institute, Cary, NC, USA).

## 3 Results

### 3.1 Patient Characteristics

**FIGURE 1:**
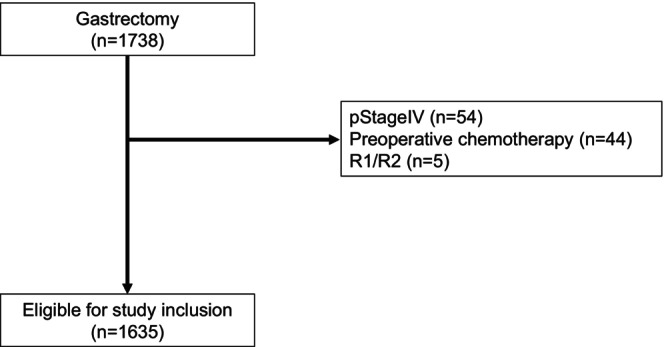
Flowchart of patient selection in the study.

A total of 1738 patients who underwent gastrectomy were initially screened from our database. After applying the inclusion criteria, 1635 eligible patients were included in the analysis. The CONSORT diagram is presented in Figure 1.

**FIGURE 2:**
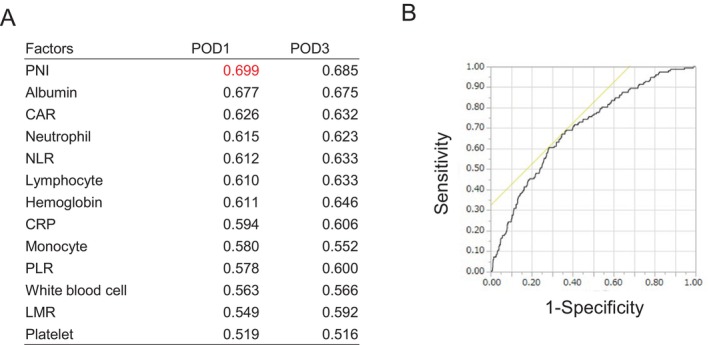
(A) Receiver operating characteristic (ROC) curve analysis and AUC for 13 variables: WBC, neutrophil, lymphocyte, monocyte, and platelet counts, CRP, hemoglobin, and albumin levels, NLR, LMR, CAR, PLR, and PNI on postoperative (POD)1 and POD3. (B) ROC of PNI on POD1, which attained the highest AUC (0.699). AUC, area under the curve; CAR, CRP‐to‐albumin ratio; CRP, C‐reactive protein; LMR, lymphocyte‐to‐monocyte ratio; NLR, neutrophil‐to‐lymphocyte ratio; PLR, platelet‐to‐lymphocyte ratio; PNI, prognostic nutritional index; POD, postoperative day; ROC, receiver operating characteristic curve; WBC, white blood cell.

The ROC curve analysis and the area under the curve (AUC) for the following 13 variables on POD1 and POD3 are shown in Figure 2A,B: WBC, neutrophil, lymphocyte, monocyte, and platelet counts, CRP, hemoglobin, and albumin levels, NLR, LMR, CAR, PLR, and PNI. The PNI on POD1 attained the highest AUC (0.699). When the cutoff value of PNI on POD1 was defined as 35 on the basis of the ROC curve analysis, the sensitivity and specificity of PNI on POD1 were 67.1% and 65.3%, respectively.

The association between low and high PNI on POD1 groups and clinicopathological factors is presented in Table 1. The low PNI on POD1 group included significantly more patients with older age (*p* < 0.001), female sex (*p* = 0.003), undifferentiated type (*p* = 0.018), lymphatic and vessel invasion (both *p* < 0.001), advanced cT stage (*p* < 0.001), advanced cN stage (*p* < 0.001), advanced cStage (*p* < 0.001), advanced pT stage (*p* < 0.001), advanced pN stage (*p* < 0.001), advanced pStage (*p* < 0.001), and adjuvant chemotherapy (*p* < 0.001).

**TABLE 1:** Patient characteristics between low and high PNI groups on POD1.

	*n* = 1635	PNI (POD1)				
		Low (< 35), *n* = 506	%	High (≥ 35), *n* = 1129	%	*p*
Age (years), median (range)	67 (25–93)	71 (25–93)		66 (27–90)		< 0.001
Sex						
Male	1115	319	63.0	796	70.5	0.003
Female	520	187	37.0	333	29.5	
Histological type						
Differentiated type	883	251	49.6	632	56.0	0.017
Undifferentiated type	752	255	50.4	497	44.0	
Lymphatic invasion						
0	1031	275	54.3	756	67.0	< 0.001
1	604	231	45.7	373	33.0	
Vascular invasion						
0	1098	264	52.2	834	73.9	< 0.001
1	537	242	47.8	295	26.1	
cT						
T1	1026	214	42.3	812	71.9	< 0.001
T2	293	114	22.5	179	15.9	
T3	182	94	18.6	88	7.8	
T4	134	84	16.6	50	4.4	
cN						
N0	1241	316	62.5	925	81.9	< 0.001
N+	394	190	37.5	204	18.1	
cStage						
Stage I	1134	259	51.2	875	77.5	< 0.001
Stage II	286	123	24.3	163	14.4	
Stage III	201	117	23.1	84	7.4	
Stage IV	14	7	1.4	7	0.6	
pT						
T1	982	204	40.3	778	68.9	< 0.001
T2	197	67	13.2	130	11.5	
T3	272	133	26.3	139	12.3	
T4	184	102	20.2	82	7.3	
pN						
N0	1089	279	55.1	810	71.7	< 0.001
N1	242	86	17.0	156	13.8	
N2	159	63	12.5	96	8.5	
N3	145	78	15.4	67	5.9	
pStage						
Stage I	1057	235	46.4	822	72.8	< 0.001
Stage II	307	133	26.3	174	15.4	
Stage III	271	138	27.3	133	11.8	
Stage IV	0	0	0.0	0	0.0	
Adjuvant treatment						
Present	374	155	30.6	219	19.4	< 0.001
Absent	1261	351	69.4	910	80.6	

Abbreviations: cStage, clinical stage; PNI, prognostic nutritional index; POD, postoperative day; pStage, pathological stage.

The association of perioperative factors with the low and high PNI on POD1 groups is presented in Table 2. The low PNI on POD1 group had significantly more patients who underwent open surgery (*p* < 0.001) or total gastrectomy (*p* < 0.001). The operative time was significantly longer in the low PNI on POD1 group than in the high PNI on POD1 group (*p* < 0.001). Blood loss was significantly greater in the low PNI on POD1 group (*p* < 0.001). The postoperative hospital stay and total hospital stay were significantly longer in the low PNI on POD1 group than in the high PNI on POD1 group (both *p* < 0.001). With respect to complications, the low PNI on POD1 group comprised significantly more patients with Clavien–Dindo grade ≤ III complications (*p* = 0.004). In particular, the rates of surgical site infection and pneumonia were significantly higher in the low PNI on POD1 group (*p* = 0.017 and *p* = 0.010, respectively).

**TABLE 2:** Intra‐ and postoperative factors between low and high PNI groups on POD1.

	*n* = 1635	PNI (POD1)				
		Low (< 35), *n* = 506	%	High (≥ 35), *n* = 1129	%	*p*
Approach						
Open	627	267	52.8	360	31.9	< 0.001
Laparoscopic	1008	239	47.2	769	68.1	
Procedure						
Total gastrectomy	430	207	40.9	223	19.8	< 0.001
Distal or proximal gastrectomy	1205	299	59.1	906	80.2	
Operative time (min), median (range)	235 (86–620)	248 (95–613)		230 (86–620)		< 0.001
Blood loss (mL)	50 (0–2025)	198 (0–1950)		25 (0–2025)		< 0.001
Postoperative hospital stay (days)	11 (5–160)	12 (6–160)		10 (5–88)		< 0.001
Total hospital stay (days)	15 (7–170)	17 (8–170)		14 (7–102)		< 0.001
Clavien–Dindo grade ≤ III complications, yes	53	26	5.1	27	2.4	0.004
Abdominal abscess	12	6	1.2	6	0.5	0.152
Anastomotic leakage	10	2	0.4	8	0.7	0.453
Pancreatic fistula	7	4	0.8	3	0.3	0.133
Bleeding	7	1	0.2	6	0.5	0.339
Bowel obstruction	6	3	0.6	3	0.3	0.312
Surgical site infection	5	4	0.8	1	0.1	0.018
Pneumonia	3	3	0.6	0	0.0	0.010
Anastomotic stenosis	3	1	0.2	2	0.2	0.929
Delayed gastric emptying	0	0	0.0	0	0.0	—

Abbreviations: PNI, prognostic nutritional index; POD, postoperative day.

### 3.2 Survival

**FIGURE 3:**
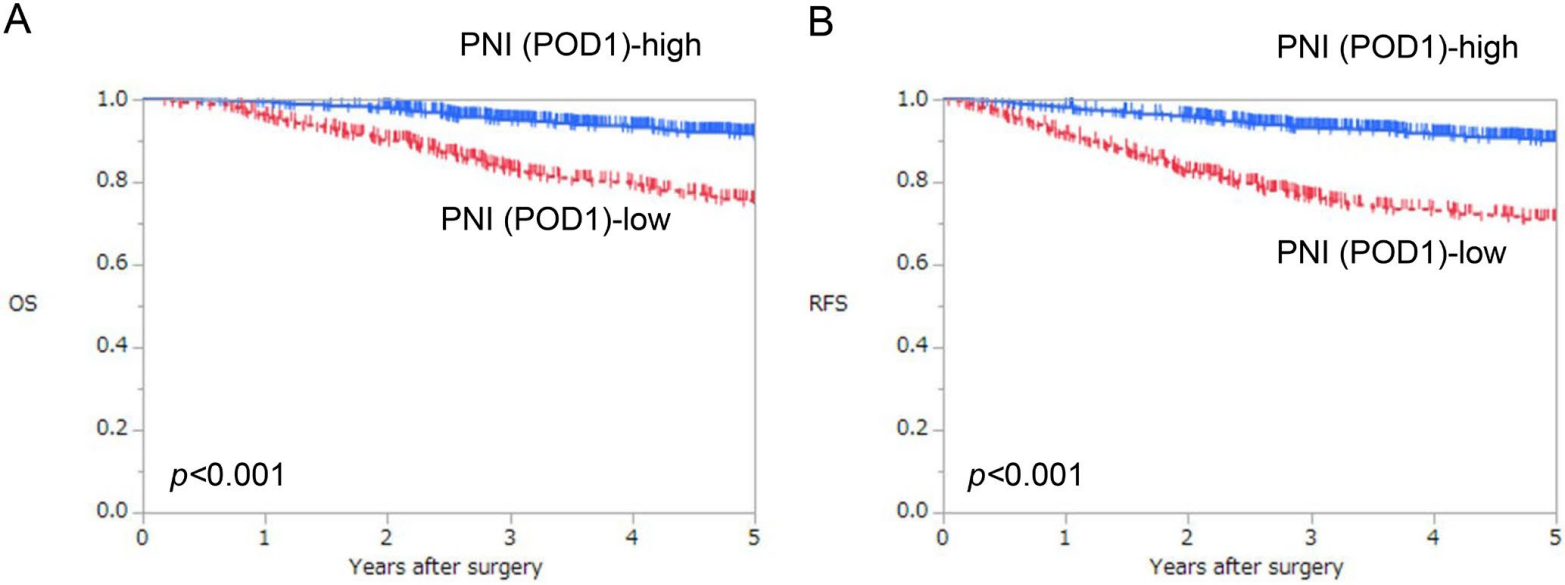
Comparison of survival curves between high and low PNI on POD1 for OS and RFS. (A) OS and (B) RFS. OS, overall survival; PNI, prognostic nutritional index; POD, postoperative day; RFS, recurrence‐free survival.

The median follow‐up period in all patients was 60 months. The 5‐year OS rates in the high and low PNI on POD1 groups were 91.4% and 75.1%, respectively. OS was significantly poorer in the low PNI on POD1 group than in the high PNI on POD1 group (log‐rank test, *p* < 0.001; Figure 3A).

The 5‐year RFS rates in the high and low PNI on POD1 groups were 90.1% and 71.0%, respectively. RFS was significantly poorer in the low PNI on POD1 group than in the high PNI on POD1 group (log‐rank test, *p* < 0.001; Figure 3B). The low PNI on POD1 group showed significantly more recurrence patterns with respect to peritoneal dissemination, lymph node, and liver, bone, and brain metastases than the high PNI on POD1 group (*p* < 0.001, *p* = 0.003, *p* < 0.001, *p* < 0.001, and *p* = 0.035, respectively) (Table 3).

**TABLE 3:** Recurrence type between low and high PNI groups on POD1.

	*n* = 1635	PNI (POD1)				
		Low (< 35), *n* = 506	%	High (≥ 35), *n* = 1129	%	*p*
Recurrence	152	90	17.8	62	5.5	< 0.001
Peritoneal dissemination	59	37	7.3	22	1.9	< 0.001
Lymph nodes	28	16	3.2	12	1.1	0.003
Liver	25	17	3.4	8	0.7	< 0.001
Lung	15	7	1.4	8	0.7	0.186
Bone	17	12	2.4	5	0.4	< 0.001
Local	7	3	0.6	4	0.4	0.495
Brain	2	2	0.4	0	0.0	0.035
Others	10	5	1.0	5	0.4	0.191

Abbreviations: PNI, prognostic nutritional index; POD, postoperative day.

**FIGURE 4:**
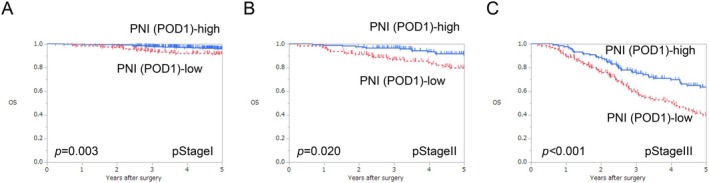
Comparison of survival curves between high and low PNI on POD1 for OS according to pStage. (A) pStage I, (B) pStage II, (C) pStage III. OS, overall survival; PNI, prognostic nutritional index; POD, postoperative day.

Subgroup analysis according to pStage revealed that OS in the low PNI on POD1 group was significantly poorer than that in the high PNI on POD1 group in each pStage (log‐rank test, *p* = 0.003, *p* = 0.020, and *p* < 0.001, respectively; Figure 4A–C). RFS in the low PNI on POD1 group was significantly poorer than that in the high PNI on POD1 group in each pStage (log‐rank test, *p* = 0.002, *p* = 0.003, and *p* < 0.001, respectively; Figure S1A–C).

Multivariate Cox analysis revealed that low PNI on POD1 was a significantly independent prognostic factor for poor OS, in addition to older age, undifferentiated type, vascular invasion, total gastrectomy, pT, and pN (*p* = 0.002; Table 4). The adjusted hazard ratio (HR) for OS was 1.57 (95% confidence incidence [CI]: 1.19–2.09) in the low PNI on POD1 group. Similarly, with respect to RFS, multivariate Cox analysis showed that low PNI on POD1 was a significantly independent prognostic factor for poor RFS, in addition to older age, vascular invasion, total gastrectomy, pT, and pN (*p* < 0.001) (Table 4). The adjusted HR for RFS in the low PNI on POD1 group was 1.70 (95% CI: 1.31–2.22).

**TABLE 4:** Multivariate Cox analysis of OS and RFS.

Variables	Categories	HR (95% CI)	*p*
OS			
Age (years)	> 66/66 ≥	2.17 (1.62–2.93)	< 0.001
Sex	Male/female	1.03 (0.77–1.41)	0.835
Histological type	Undifferentiated/differentiated	1.50 (1.13–1.99)	0.005
Lymphatic invasion	1/0	1.05 (0.77–1.45)	0.747
Vascular invasion	1/0	1.62 (1.16–2.26)	0.004
Gastrectomy	TG	1.38 (1.04–1.82)	0.024
pT	T3–4/1–2	2.32 (1.63–3.33)	< 0.001
pN	N1–3/0	2.82 (1.95–4.09)	< 0.001
Adjuvant treatment	No/yes	1.32 (0.96–1.81)	0.089
Postoperative complications (Clavien–Dindo grade ≤ III)	Yes/no	1.51 (0.81–2.56)	0.179
PNI‐POD1	Low/high	1.57 (1.19–2.09)	0.0017
RFS			
Age (years)	> 66/66 ≥	1.88 (1.44–2.48)	< 0.001
Sex	Male/female	1.13 (0.86–1.50)	0.396
Histological type	Undifferentiated/differentiated	1.29 (0.99–1.67)	0.056
Lymphatic invasion	1/0	1.06 (0.79–1.41)	0.707
Vascular invasion	1/0	1.61 (1.19–2.20)	0.002
Gastrectomy	TG	1.50 (1.16–1.94)	0.002
pT	T3–4/1–2	2.18 (1.57–3.04)	< 0.001
pN	N1–3/0	2.99 (2.12–4.24)	< 0.001
Adjuvant treatment	No/yes	1.16 (0.86–1.55)	0.325
Postoperative complications (Clavien–Dindo grade ≤ III complications)	Yes/no	1.29 (0.71–2.14)	0.379
PNI‐POD1	Low/high	1.70 (1.31–2.22)	< 0.001

Abbreviations: OS, overall survival; PNI, prognostic nutritional index; POD, postoperative day; RFS, recurrence‐free survival.

## 4 Discussion

To the best of our knowledge, no previous study has reported on prognostic markers that take preoperative nutritional status, surgical stress, and postoperative inflammatory response into account in patients with cancer. The present study is the first to report on postoperative PNI as a prognostic marker. This study revealed that low postoperative PNI was significantly associated with poor prognosis in terms of both RFS and OS in patients with gastric cancer after surgery. Moreover, low postoperative PNI was an independent poor prognostic factor for both RFS and OS in patients with gastric cancer after surgery.

Preoperative PNI is generally utilized as an indicator for nutritional assessment and prognosis prediction and is reportedly useful in predicting the onset of postoperative complications in surgical patients with various types of cancer before surgery [2]. Although postoperative inflammatory response related to surgical invasiveness is considered to be associated with prognosis, preoperative PNI does not account for the impact of surgical invasiveness on the postoperative inflammatory response [6]. In contrast, postoperative PNI reflects the surgical invasiveness and postoperative inflammatory response associated with complications. Postoperative systemic inflammation has recently been suggested to potentially have an adverse impact on the prognosis of patients with cancer [6]. Surgical stress induces the production of inflammatory cytokines to restore homeostasis; however, excessive surgical stress may lead to the overproduction of inflammatory cytokines, resulting in the proliferation and migration of cancer cells [11, 12, 13]. Surgical stress also causes a decline in albumin levels due to intraoperative loss and increased catabolism. The greater the surgical stress (e.g., open surgery, total gastrectomy, prolonged surgery, substantial blood loss), the more pronounced the decrease in albumin levels. Additionally, the postoperative inflammatory response increases with surgical stress and postoperative complications, resulting in an elevated neutrophil count and a decreased lymphocyte count. Therefore, the postoperative PNI can serve as an indicator reflective of both the preoperative nutritional status and the postoperative inflammatory response caused by surgical stress and complications. In this study, the PNI on POD1 was significantly lower in the open group than in the laparoscopic group at pStages I, II, and III (each *p* < 0.001; Figure S2A–C). Additionally, the total gastrectomy group had a significantly lower PNI on POD1 than the distal and proximal gastrectomy group (each *p* < 0.001; Figure S3A–C).

Comparisons of preoperative PNI and postoperative PNI in the present study indicated that the AUC for postoperative PNI (0.699) was higher than the AUC for preoperative PNI (0.621); furthermore, the sensitivity and specificity of preoperative PNI were 39.5% and 80.7%, respectively (Figure S4). The Kaplan–Meier curves for the four groups categorized according to preoperative PNI and postoperative PNI (POD1) are shown in Figure S5A,B. Interestingly, our results showed that the group with high preoperative PNI and low postoperative PNI tended to have worse prognosis than the group with low preoperative PNI and high postoperative PNI (OS: *p* = 0.079, RFS: *p* = 0.015; Figure S6A,B), suggesting that a low postoperative PNI after surgical invasion may lead to poor prognosis even in populations with high preoperative PNI. Conversely, in populations with low preoperative PNI, prognosis can possibly be improved by providing sufficient nutritional interventions before surgery and minimizing surgical invasion, resulting in a high postoperative PNI. Compared with preoperative PNI, postoperative PNI may serve as a prognostic indicator that better reflects surgical invasion and other factors. Since PNI is calculated using weighted values of albumin levels and lymphocyte counts, its value on POD1 is likely to reflect the impact of surgical stress. Therefore, we also evaluated the relative association between PNI on POD1 and other inflammatory and nutritional markers. The PNI level on POD1 was significantly associated with albumin, CAR, NLR, hemoglobin, CRP, PLR, white blood cell count, and LMR, but not with platelet count (Table S1).

Increased postoperative inflammatory response is reportedly associated with poor prognosis [5]. The mechanism underlying the association between increased postoperative inflammatory response and poor prognosis may involve systemic inflammatory reactions that stimulate the production of various inflammatory cytokines such as interleukin (IL)‐6 and IL‐1β, similar to growth factors like epidermal growth factor and vascular endothelial growth factor [13, 14]. These molecules promote the proliferation and migration of cancer cells [11, 12, 13]. Additionally, cancer cells can exploit inflammatory pathways—such as selectin–ligand interactions, matrix metalloproteinase (MMP) activity, and chemokine signaling—to facilitate their dissemination and metastatic potential. This hijacking of immune‐related processes may represent a strategy by which tumors evade host immune surveillance and promote disease progression [13]. Thus, systemic inflammation is associated with host immunosuppression, which in turn facilitates cancer growth and metastasis [15].

Postoperative complications are the primary cause of increased postoperative inflammatory response. In this study, we demonstrated for the first time that postoperative PNI correlated with the occurrence of postoperative complications and may serve as a prognostic indicator. However, the causal relationship is unclear. Specifically, it is uncertain whether a low PNI predisposes patients to complications, or whether early postoperative complications lead to systemic inflammation, resulting in a subsequent decline in PNI. We believe that both mechanisms are plausible. Previous studies have shown that a low preoperative PNI reflecting poor nutritional status is associated with a higher risk of postoperative complications [2, 3, 4]. Based on this, we primarily consider that a low postoperative PNI, which also reflects nutritional status at the time of surgery, may contribute to the development of complications. Conversely, it is also possible that early postoperative complications lead to systemic inflammation, thereby reducing the PNI observed on POD1. The latter mechanism is supported by the following analytical results. We evaluated postoperative PNI levels on POD1 and POD3 in patients with and without postoperative complications. As shown in Table 2, the PNI on POD1 was significantly associated with postoperative complications (*p* < 0.001). Furthermore, the PNI levels on both POD1 and POD3 were significantly lower in the group with postoperative complications compared to those without complications (*p* = 0.009 and *p* < 0.001, respectively; Table S2). Although PNI levels declined from POD1 to POD3 in both groups, the decrease was more pronounced in patients who experienced complications. These findings suggest that postoperative PNI may reflect the presence and severity of postoperative complications. Additionally, we analyzed the incidence of complications among four groups based on preoperative and postoperative PNI status: high preoperative PNI and high postoperative PNI (HH), high preoperative PNI and low postoperative PNI (HL), low preoperative PNI and high postoperative PNI (LH), and low preoperative PNI and low postoperative PNI (LL). To isolate the effect of postoperative PNI, we compared the HH and HL groups, which shared similarly good preoperative nutritional status. The postoperative complication rate was higher in the HL group compared to the HH group (5.4% vs. 2.4%, respectively; Table S3). These results indicate that, even when preoperative nutritional status was good, a low postoperative PNI may be associated with the occurrence of complications.

Postoperative CRP levels have been shown by previous studies to be useful in predicting both postoperative complications and prognosis [16, 17, 18, 19, 20]. In this study, ROC curve analysis revealed that postoperative PNI achieved a higher AUC than postoperative CRP levels (Figure 2). This is likely because postoperative CRP levels reflect only the postoperative inflammatory response, whereas postoperative PNI integrates preoperative nutritional status, surgical stress, and postoperative inflammatory response. Therefore, postoperative PNI is considered a more useful biomarker.

Two key factors are crucial for improving postoperative PNI in prognosis: implementing preoperative nutritional interventions that improve prognosis [21, 22, 23, 24] and minimizing surgical stress. Preoperative nutritional support was provided through enteral nutrition or total parenteral nutrition. We examined patients who had been hospitalized at least 14 days before surgery to receive preoperative nutrition. Among 1635 patients, 50 (3.1%) were hospitalized 14 days prior to surgery. The proportions of patients who received preoperative nutritional interventions across the four PNI‐based groups were as follows: 2.2% (21/959), 2.7% (6/224), 5.9% (10/170), and 4.6% (13/282) for HH, HL, LH, and LL groups, respectively. The ratio of preoperative nutritional interventions in the LH group was the highest (5.9%). Of the 23 patients with low preoperative PNI who received preoperative nutritional intervention, 10 (43.5%) achieved high postoperative PNI. Among the low preoperative PNI groups (LH and LL), the OS and RFS in the LH group were better compared to the LL group (both *p* < 0.001; Figure S7). These findings suggest that preoperative nutritional interventions may help improve postoperative PNI (POD1), thereby potentially enhancing long‐term prognosis. Laparoscopic surgery, a minimally invasive procedure, offers advantages over open surgery in terms of reducing surgical stress as a result of its lower invasiveness. Results from previous trials underscore that laparoscopic surgery ensures oncological efficacy and is associated with a lower postoperative complication rate, reduced blood loss, and shorter operative time compared to open surgery, highlighting its minimally invasive nature [25, 26, 27, 28]. In this study, the PNI on POD1 was significantly lower in the open group than in the laparoscopic group at pStages I, II, and III (each *p* < 0.001; Figure S2A–C).

This study has some limitations. In particular, this retrospective study was conducted at a single center and lacked validation of the results. To address potential confounding factors, we performed a propensity score matching analysis. After matching, patient characteristics were well balanced between groups (Table S4). The associations between perioperative factors and PNI on POD1 remained consistent with the original analysis, except for postoperative complications (Table S5). The lack of a significant difference in postoperative complications may be attributable to the relatively small number of events. Nonetheless, both OS and RFS were significantly poorer in the low PNI on POD1 group compared to the high PNI on POD1 group (log‐rank test, *p* = 0.016 and *p* < 0.001, respectively; Figure S8). Moreover, a prospective multicenter study with a larger sample size should be conducted to further validate our findings.

In conclusion, this study showed that the postoperative PNI was a useful prognostic factor in patients with gastric cancer.

## Author Contributions

**Masaaki Yamamoto:** data curation, conceptualization, methodology, investigation, writing – original draft, writing – review and editing, visualization, project administration, validation, software, formal analysis, supervision. **Takeshi Omori:** writing – review and editing, supervision, project administration, methodology. **Yasunori Masuike:** data curation. **Naoki Shinno:** data curation. **Hisashi Hara:** data curation. **Takahito Sugase:** data curation. **Takashi Kanemura:** data curation. **Atsushi Takeno:** data curation. **Motohiro Hirao:** supervision. **Hiroshi Miyata:** supervision, data curation, project administration.

## Ethics Statement

The protocol for this research project conformed to the provisions outlined in the Declaration of Helsinki and was approved by the Ethics Review Committee of Osaka International Cancer Institute (approval number #23153).

## Consent

Informed consent was obtained from all participants.

## Conflicts of Interest

The authors declare no conflicts of interest.

## Supporting information


**Figure S1.** Comparison of survival curves between low and high PNI on POD1 for recurrence‐free survival according to pStage.

**Figure S2.** PNI on POD1 between open and laparoscopic gastrectomy according to pStage.

**Figure S3.** PNI on POD1 between total and (distal and proximal) gastrectomy according to pStage.

**Figure S4.** ROC of preoperative PNI.

**Figure S5.** Comparison of survival curves between high and low PNI before and after surgery for OS and RFS.

**Figure S6.** Comparison of survival curves between high and low PNI before and after surgery for OS and RFS.

**Figure S7.** Comparison of survival curves for OS and RFS between high and low postoperative PNI in patients with low preoperative PNI.

**Figure S8.** Comparison of survival curves for OS and RFS between high and low PNI on POD1 after matching.

**Table S1.** Association between PNI on POD1 and other clinical markers.
**Table S2.** Association between postoperative complications and PNI.
**Table S3.** Comparison of postoperative complications among HH, HL, LH, and LL groups.
**Table S4.** Patient characteristics between low and high PNI groups on POD1, before and after matching.
**Table S5.** Intraoperative and postoperative factors between low and high PNI groups on POD1, before and after matching.
