# Supplementary figures and images for: Postoperative Prognostic Nutritional Index as a Useful Prognostic Factor in Patients With Gastric Cancer
Source: Ann Gastroenterol Surg. 2025 Jun 19;9(6):1181–90. doi: 10.1002/ags3.70057 (PMC12586946; doi:10.1002/ags3.70057)

# Supplementary Figure 1

A

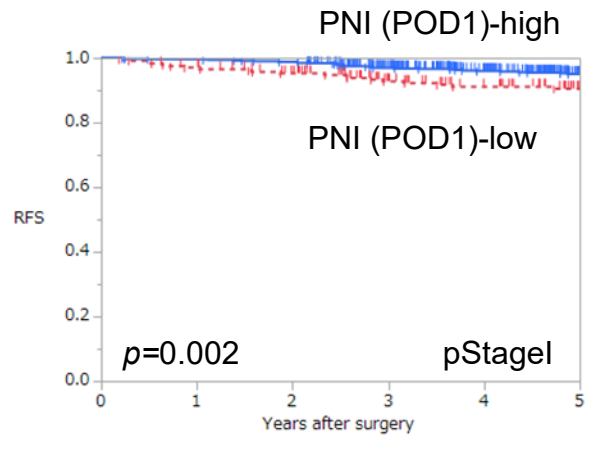

B

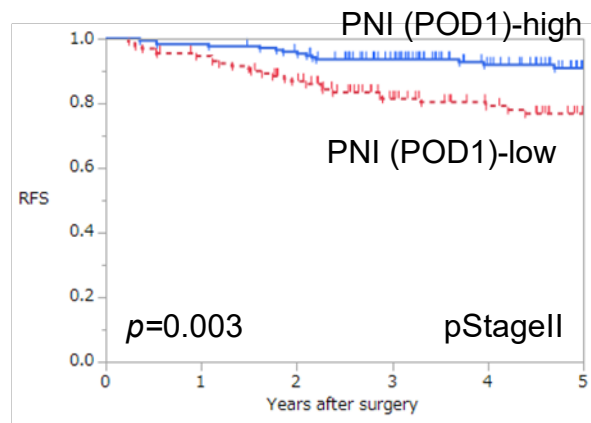

C

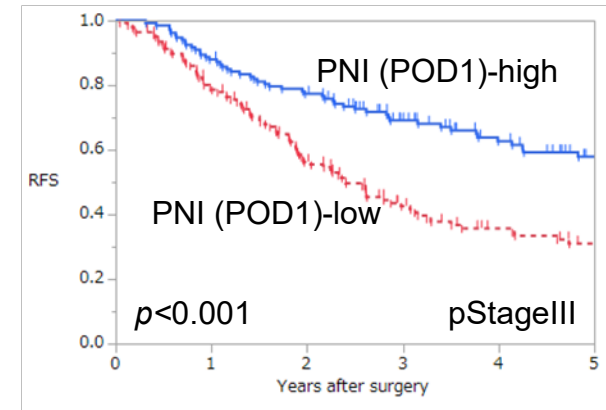

Supplement: Supplementary file 1 — Figure S1. Comparison of survival curves between low and high PNI on POD1 for recurrence‐free survival according to pStage. [file AGS3-9-1181-s008.pdf]

Supplementary  
Figure 2

A

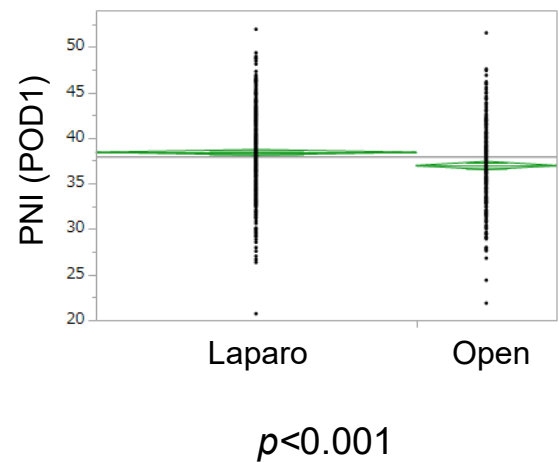

B

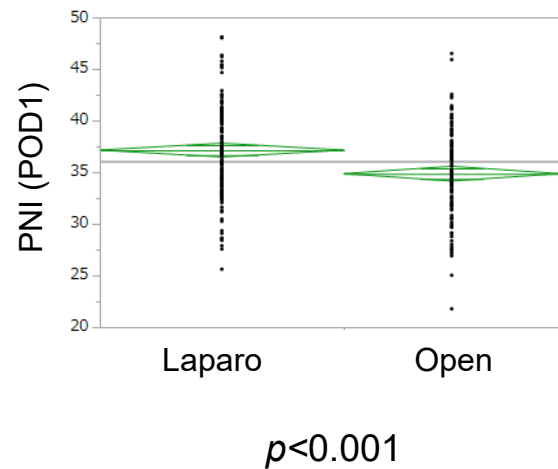

C

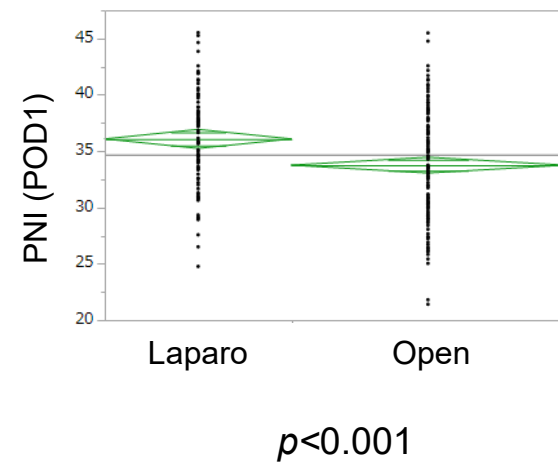

Supplement: Supplementary file 2 — Figure S2. PNI on POD1 between open and laparoscopic gastrectomy according to pStage. [file AGS3-9-1181-s006.pdf]

Supplementary  
Figure 3

A

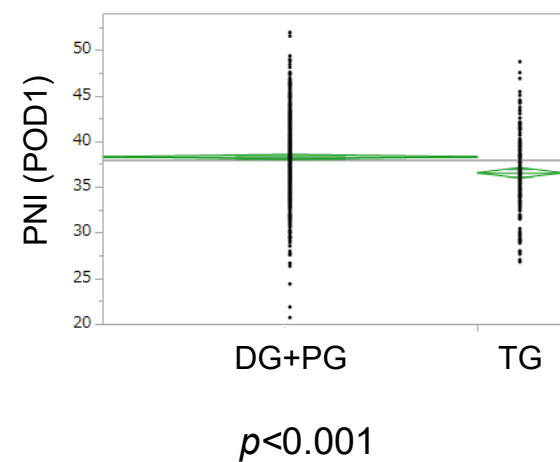

B

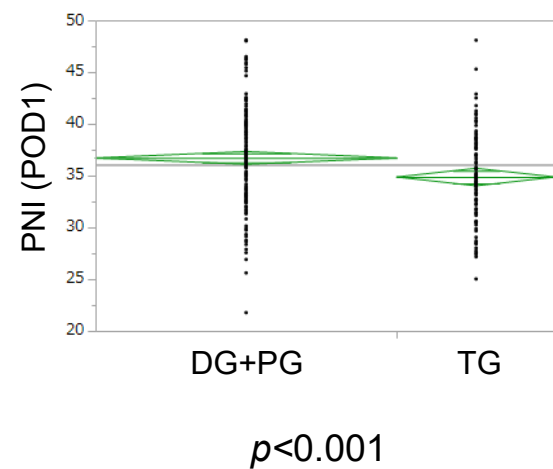

C

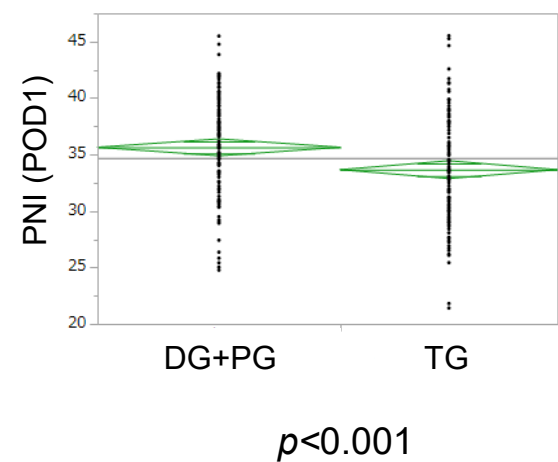

Supplement: Supplementary file 3 — Figure S3. PNI on POD1 between total and (distal and proximal) gastrectomy according to pStage. [file AGS3-9-1181-s002.pdf]

Supplementary  
Figure 4

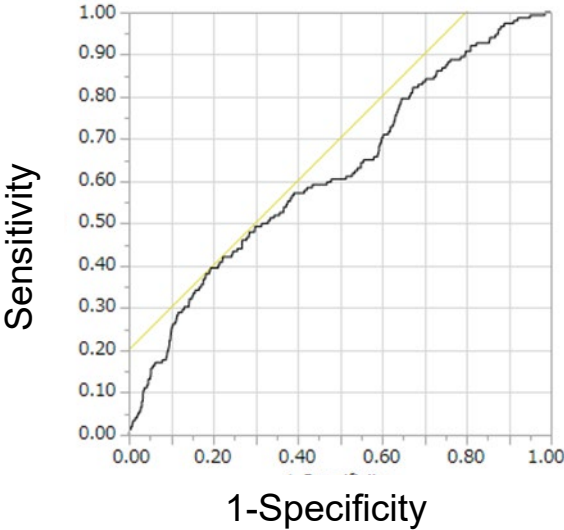

Supplement: Supplementary file 4 — Figure S4. ROC of preoperative PNI. [file AGS3-9-1181-s009.pdf]

Supplementary  
Figure 5

A

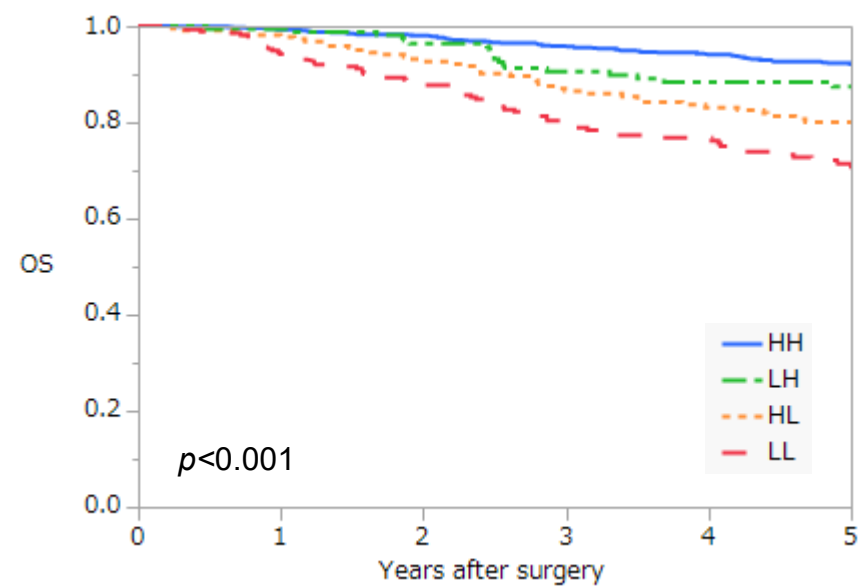

B

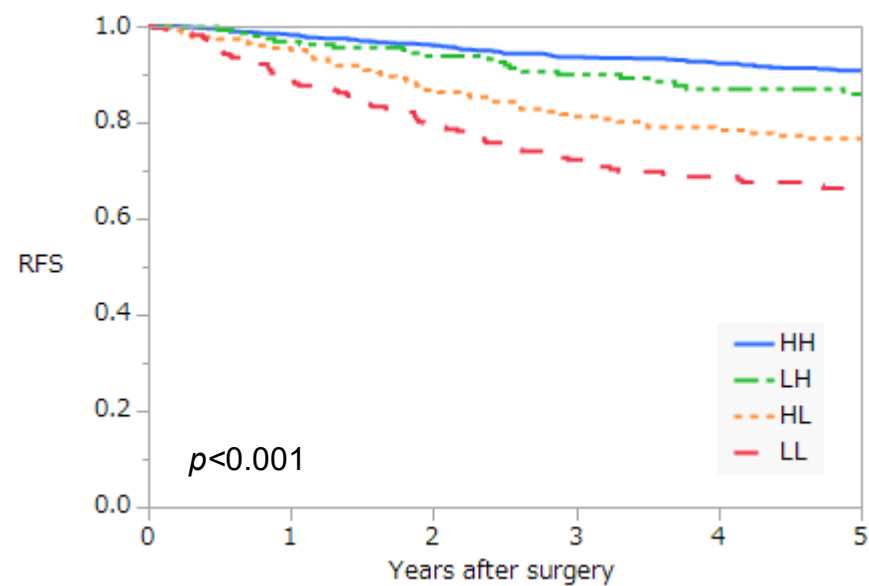

Supplement: Supplementary file 5 — Figure S5. Comparison of survival curves between high and low PNI before and after surgery for OS and RFS. [file AGS3-9-1181-s001.pdf]

# Supplementary Figure6

A

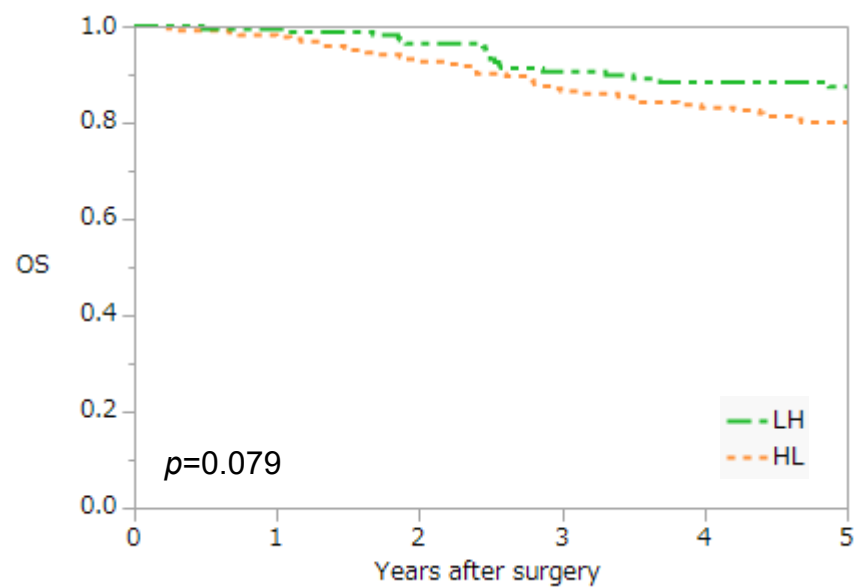

B

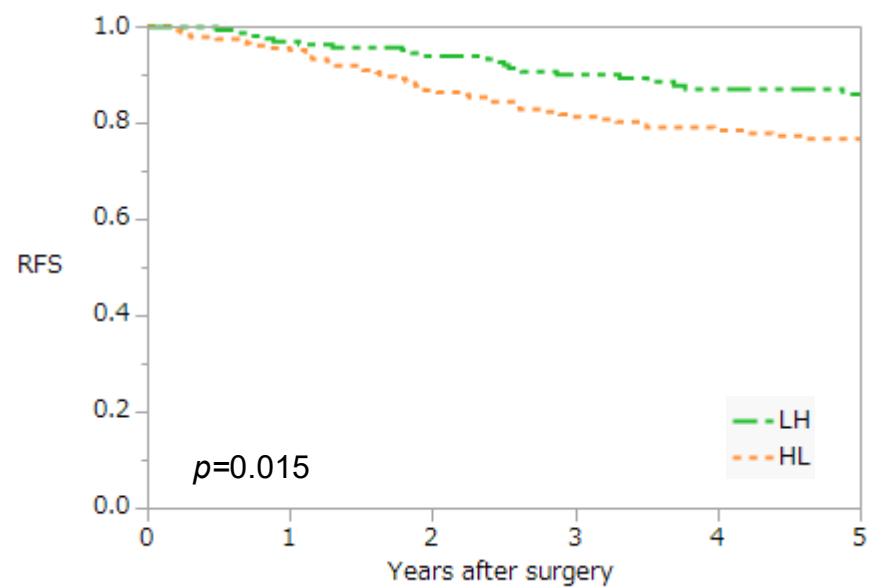

Supplement: Supplementary file 6 — Figure S6. Comparison of survival curves between high and low PNI before and after surgery for OS and RFS. [file AGS3-9-1181-s007.pdf]

# Supplementary Figure7

A

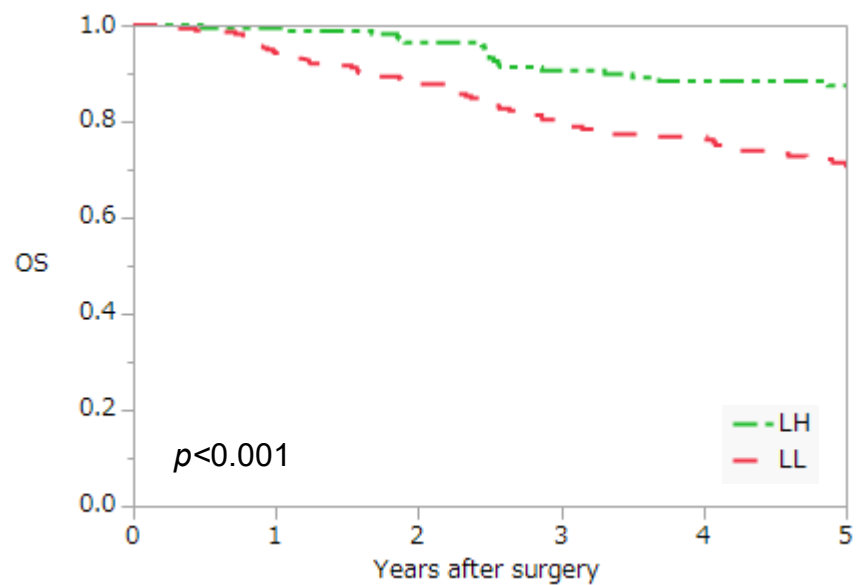

B

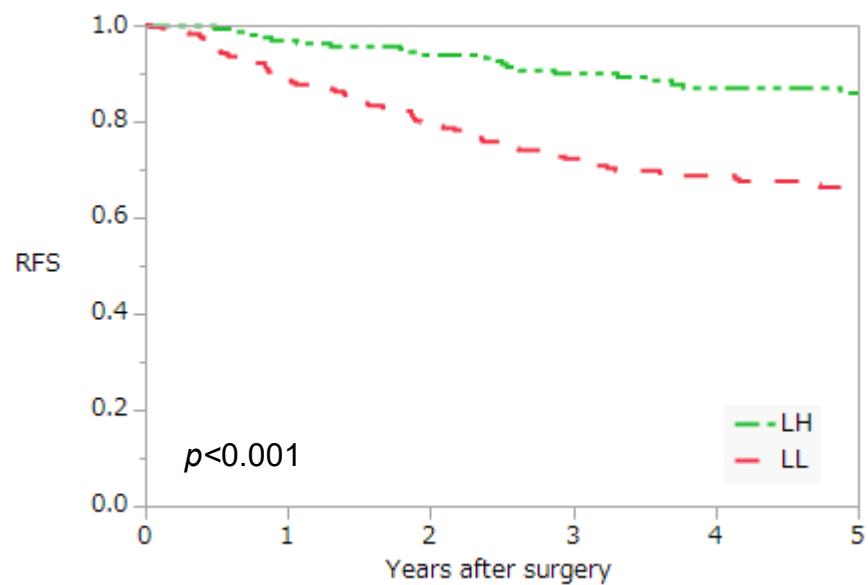

Supplement: Supplementary file 7 — Figure S7. Comparison of survival curves for OS and RFS between high and low postoperative PNI in patients with low preoperative PNI. [file AGS3-9-1181-s005.pdf]

# Supplementary Figure8

A

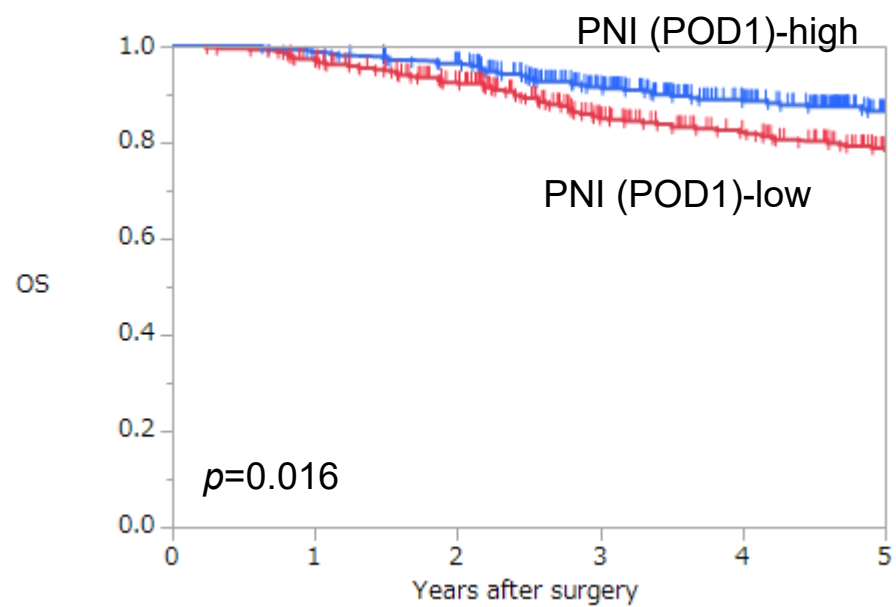

B

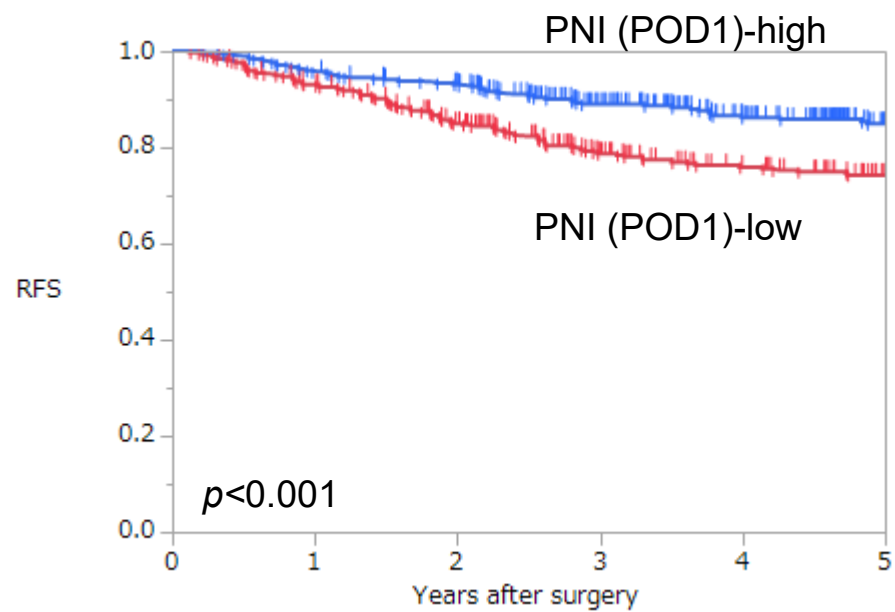

Supplement: Supplementary file 8 — Figure S8. Comparison of survival curves for OS and RFS between high and low PNI on POD1 after matching. [file AGS3-9-1181-s003.pdf]
