# Supplementary material for: Postoperative Prognostic Nutritional Index as a Useful Prognostic Factor in Patients With Gastric Cancer
Source: Ann Gastroenterol Surg. 2025 Jun 19;9(6):1181–90. doi: 10.1002/ags3.70057 (PMC12586946; doi:10.1002/ags3.70057)
Supplement: Supplementary file 9 — Table S1. Association between PNI on POD1 and other clinical markers. Table S2. Association between postoperative complications and PNI. Table S3. Comparison of postoperative complications among HH, HL, LH, and LL groups. Table S4. Patient characteristics between low and high PNI groups on POD1, before and after matching. Table S5. Intraoperative and postoperative factors between low and high PNI groups on POD1, before and after matching. [file AGS3-9-1181-s004.docx]

**TABLE S1** Association between PNI on POD1 and other clinical markers

|  | PNI (POD1) | | |
| --- | --- | --- | --- |
|  | low (< 35) *n*=506 | high (≧ 35) *n*=1129 | *p* |
| Albumin, g/dL | 2.8 (1.6-3.3) | 3.3 (2.4-4.3) | <0.001 |
| CAR | 2.1 (0.1-8.8) | 1.5 (0.1-5.8) | <0.001 |
| NLR | 8.0 (2.0-37.1) | 6.2 (2.1-30.4) | <0.001 |
| Hemoglobin, g/dL | 11.3 (7.5-15.7) | 12.7 (7.1-17.1) | <0.001 |
| CRP, mg/dL | 5.8 (0.2-22.4) | 5.0 (0.2-17.6) | <0.001 |
| PLR | 201 (55-757) | 154 842-664) | <0.001 |
| White blood cell, /μL | 8885 (3770-23920) | 9760 (4290-24020) | <0.001 |
| LMR | 2.1 (0.6-9.8) | 2.3 (0.6-8.4) | <0.001 |
| Platelet, x10^4^/μL | 18.6 (4.5-51.0) | 19.1 (7.3-40.6 | 0.123 |

Abbreviations: CRP, C-reactive protein; NLR, neutrophil-to-lymphocyte ratio; LMR, lymphocyte-to-monocyte ratio; CAR, CRP-to-albumin ratio; PLR, platelet-to-lymphocyte ratio; PNI, prognostic nutritional index; POD, postoperative day.

**TABLE S2**  Association between postoperative complications and PNI

|  | Clavien–Dindo grade ≤III complications | |  |
| --- | --- | --- | --- |
|  | yes, *n*=53 | no, *n*=1582 | *p* |
| PNI (POD1) | 35.1 (21.4-44.9) | 37.3 (20.7-51.9) | 0.009 |
| PNI (POD3) | 32.5 (17.9-45.9) | 36.4 (21.6-57.6) | <0.001 |

Abbreviations: PNI, prognostic nutritional index; POD, postoperative day.

**TABLE S3**  Comparison of postoperative complications among HH, HL, LH, and LL groups

|  |  | Clavien–Dindo grade ≤III complications , yes | |
| --- | --- | --- | --- |
|  | *n*=1635 | *n*=53 | ％ |
| HH | 959 | 23 | 2.4 |
| HL | 224 | 12 | 5.4 |
| LH | 170 | 4 | 2.4 |
| LL | 282 | 14 | 5.0 |

Abbreviations: PNI, prognostic nutritional index; POD, postoperative day; HH, high preoperative PNI and low postoperative PNI; LH, low preoperative PNI and high postoperative PNI; HL, high preoperative PNI and low postoperative PNI; LL, low preoperative PNI and low postoperative PNI.

**TABLE S4**  Patient characteristics between low and high PNI groups on POD1, before and after matching

|  |  | Before matching | | | | |  | After matching | | | | |
| --- | --- | --- | --- | --- | --- | --- | --- | --- | --- | --- | --- | --- |
|  |  | PNI (POD1) | | | | |  | PNI (POD1) | | | | |
|  | *n*=1635 | low (< 35) *n*=506 | % | high (≧ 35) *n*=1129 | % | *p* |  | low (< 35) *n*=424 | % | high (≧ 35) *n*=424 | % | *p* |
| Age (years), median (range) | 67 (25-93) | 71 (25-93) |  | 66 (27-90) |  | <0.001 |  | 69.5 (25-91) |  | 70 (38-90) |  | 0.512 |
| Sex |  |  |  |  |  | 0.003 |  |  |  |  |  | 0.343 |
| Male | 1115 | 319 | 63.0 | 796 | 70.5 |  |  | 277 | 65.3 | 290 | 68.4 |  |
| Female | 520 | 187 | 37.0 | 333 | 29.5 |  |  | 147 | 34.7 | 134 | 31.6 |  |
| Histological type | | |  |  |  | 0.017 |  |  |  |  |  | 0.680 |
| Differentiated type | 883 | 251 | 49.6 | 632 | 56.0 |  |  | 219 | 51.7 | 225 | 53.1 |  |
| Undifferentiated type | 752 | 255 | 50.4 | 497 | 44.0 |  |  | 205 | 48.3 | 199 | 46.9 |  |
| Ly |  |  |  |  |  | <0.001 |  |  |  |  |  | 0.144 |
| 0 | 1031 | 275 | 54.3 | 756 | 67.0 |  |  | 236 | 55.7 | 257 | 60.6 |  |
| 1 | 604 | 231 | 45.7 | 373 | 33.0 |  |  | 188 | 44.3 | 167 | 39.4 |  |
| V |  |  |  |  |  | <0.001 |  |  |  |  |  | 0.017 |
| 0 | 1098 | 264 | 52.2 | 834 | 73.9 |  |  | 239 | 56.4 | 273 | 64.4 |  |
| 1 | 537 | 242 | 47.8 | 295 | 26.1 |  |  | 185 | 43.6 | 151 | 35.6 |  |
| cT |  |  |  |  |  | <0.001 |  |  |  |  |  | 0.809 |
| T1 | 1026 | 214 | 42.3 | 812 | 71.9 |  |  | 213 | 50.2 | 218 | 51.4 |  |
| T2 | 293 | 114 | 22.5 | 179 | 15.9 |  |  | 99 | 23.3 | 97 | 22.9 |  |
| T3 | 182 | 94 | 18.6 | 88 | 7.8 |  |  | 62 | 14.6 | 67 | 15.8 |  |
| T4 | 134 | 84 | 16.6 | 50 | 4.4 |  |  | 50 | 11.8 | 42 | 9.9 |  |
| cN |  |  |  |  |  | <0.001 |  |  |  |  |  | 0.823 |
| N0 | 1241 | 316 | 62.5 | 925 | 81.9 |  |  | 292 | 68.9 | 295 | 69.6 |  |
| N+ | 394 | 190 | 37.5 | 204 | 18.1 |  |  | 132 | 31.1 | 129 | 30.4 |  |
| cStage |  |  |  |  |  | <0.001 |  |  |  |  |  | 0.914 |
| StageI | 1134 | 259 | 51.2 | 875 | 77.5 |  |  | 251 | 59.2 | 258 | 60.8 |  |
| StageII | 286 | 123 | 24.3 | 163 | 14.4 |  |  | 99 | 23.3 | 91 | 21.5 |  |
| StageIII | 201 | 117 | 23.1 | 84 | 7.4 |  |  | 69 | 16.3 | 69 | 16.3 |  |
| StageIV | 14 | 7 | 1.4 | 7 | 0.6 |  |  | 5 | 1.2 | 6 | 1.4 |  |
| pT |  |  |  |  |  | <0.001 |  |  |  |  |  | 0.092 |
| T1 | 982 | 204 | 40.3 | 778 | 68.9 |  |  | 197 | 46.5 | 233 | 55.0 |  |
| T2 | 197 | 67 | 13.2 | 130 | 11.5 |  |  | 58 | 13.7 | 53 | 12.5 |  |
| T3 | 272 | 133 | 26.3 | 139 | 12.3 |  |  | 98 | 23.1 | 82 | 19.3 |  |
| T4 | 184 | 102 | 20.2 | 82 | 7.3 |  |  | 71 | 16.7 | 56 | 13.2 |  |
| pN |  |  |  |  |  | <0.001 |  |  |  |  |  | 0.897 |
| N0 | 1089 | 279 | 55.1 | 810 | 71.7 |  |  | 253 | 59.7 | 260 | 61.3 |  |
| N1 | 242 | 86 | 17.0 | 156 | 13.8 |  |  | 73 | 17.2 | 74 | 17.5 |  |
| N2 | 159 | 63 | 12.5 | 96 | 8.5 |  |  | 44 | 10.4 | 43 | 10.1 |  |
| N3 | 145 | 78 | 15.4 | 67 | 5.9 |  |  | 54 | 12.7 | 47 | 11.1 |  |
| pStage |  |  |  |  |  | <0.001 |  |  |  |  |  | 0.123 |
| StageI | 1057 | 235 | 46.4 | 822 | 72.8 |  |  | 227 | 53.5 | 256 | 60.4 |  |
| StageII | 307 | 133 | 26.3 | 174 | 15.4 |  |  | 99 | 23.3 | 81 | 19.1 |  |
| StageIII | 271 | 138 | 27.3 | 133 | 11.8 |  |  | 98 | 23.1 | 87 | 20.5 |  |
| StageIV | 0 | 0 | 0.0 | 0 | 0.0 |  |  | 0 | 0.0 | 0 | 0.0 |  |
| Adjuvant treatment | | |  |  |  | <0.001 |  |  |  |  |  | 0.592 |
| Present | 374 | 155 | 30.6 | 219 | 19.4 |  |  | 122 | 28.8 | 115 | 27.1 |  |
| Absent | 1261 | 351 | 69.4 | 910 | 80.6 |  |  | 302 | 71.2 | 309 | 72.9 |  |

Abbreviations: PNI, prognostic nutritional index; POD, postoperative day; cStage, clinical stage; pStage, pathological stage.

**TABLE S5** Intraoperative and postoperative factors between low and high PNI groups on POD1, before and after matching

|  |  | Before matching | | | | |  | After matching | | | | |
| --- | --- | --- | --- | --- | --- | --- | --- | --- | --- | --- | --- | --- |
|  |  | PNI (POD1) | | | | |  | PNI (POD1) | | | | |
|  | *n*=1635 | low (< 35) *n*=506 | % | high (≧ 35) *n*=1129 | % | *p* |  | low (< 35) *n*=424 | % | high (≧ 35) *n*=424 | % | *p* |
| Approach | |  |  |  |  | <0.001 |  |  |  |  |  | <0.001 |
| Open | 627 | 267 | 52.8 | 360 | 31.9 |  |  | 222 | 52.4 | 142 | 33.5 |  |
| Laparoscopic | 1008 | 239 | 47.2 | 769 | 68.1 |  |  | 202 | 47.6 | 282 | 66.5 |  |
| Procedure | |  |  |  |  | <0.001 |  |  |  |  |  | <0.001 |
| Total gastrectomy | 430 | 207 | 40.9 | 223 | 19.8 |  |  | 166 | 39.2 | 90 | 21.2 |  |
| Distal or proximal gastrectomy | 1205 | 299 | 59.1 | 906 | 80.2 |  |  | 258 | 60.8 | 334 | 78.8 |  |
| Operation time (min), median (range) | 235 (86-620) | 248 (95-613) |  | 230 (86-620) |  | <0.001 |  | 248 (95-613) |  | 230 (95-503) |  | <0.001 |
| Blood loss (ml) | 50 (0-2025) | 198 (0-1950) |  | 25 (0-2025) |  | <0.001 |  | 197.5 (0-1950) |  | 25 (0-1985) |  | <0.001 |
| Postoperative hospital stay (days) | 11 (5-160) | 12 (6-160) |  | 10 (5-88) |  | <0.001 |  | 12 (6-115) |  | 10 (6-61) |  | <0.001 |
| Total hospital stay (days) | 15 (7-170) | 17 (8-170) |  | 14 (7-102) |  | <0.001 |  | 17 (8-122) |  | 13 (7-67) |  | <0.001 |
| Complications CD III≦, yes | 53 | 26 | 5.1 | 27 | 2.4 | 0.004 |  | 19 | 4.5 | 13 | 3.1 | 0.280 |
| Abdominal abscess | 12 | 6 | 1.2 | 6 | 0.5 | 0.152 |  | 5 | 1.2 | 4 | 0.9 | 0.738 |
| Anastomotic leakage | 10 | 2 | 0.4 | 8 | 0.7 | 0.453 |  | 2 | 0.5 | 2 | 0.5 | 1.000 |
| Pancreatic fistula | 7 | 4 | 0.8 | 3 | 0.3 | 0.133 |  | 2 | 0.5 | 2 | 0.5 | 1.000 |
| Bleeding | 7 | 1 | 0.2 | 6 | 0.5 | 0.339 |  | 1 | 0.2 | 3 | 0.7 | 0.316 |
| Bowel obstruction | 6 | 3 | 0.6 | 3 | 0.3 | 0.312 |  | 2 | 0.5 | 2 | 0.5 | 1.000 |
| Surgical site infection | 5 | 4 | 0.8 | 1 | 0.1 | 0.018 |  | 3 | 0.7 | 0 | 0.0 | 0.083 |
| Pneumonia | 3 | 3 | 0.6 | 0 | 0.0 | 0.010 |  | 2 | 0.5 | 0 | 0.0 | 0.499 |
| Anastomotic stenosis | 3 | 1 | 0.2 | 2 | 0.2 | 0.929 |  | 1 | 0.2 | 1 | 0.2 | 1.000 |
| Delayed gastric emptying | 0 | 0 | 0.0 | 0 | 0.0 | - |  | 0 | 0.0 | 0 | 0.0 | - |

Abbreviations: PNI, prognostic nutritional index; POD, postoperative day.
